# Provider and patient perspectives to improve lung cancer screening with low-dose computed tomography 5 years after Medicare coverage: a qualitative study

**DOI:** 10.1186/s12875-022-01925-2

**Published:** 2022-12-20

**Authors:** Meghan C. Martinez, Cheryl D. Stults, Jiang Li

**Affiliations:** Center for Health Systems Research, Palo Alto Medical Foundation Research Institute, Sutter Health, 795 El Camino Real, Ames Building, Palo Alto, CA 94301 USA

**Keywords:** Lung cancer, Lung cancer screening, Low dose computed tomography, Health care barriers, Qualitative interviews

## Abstract

Lung cancer remains the leading cause of cancer-related deaths for both men and women in the U.S., yet uptake of preventive cancer screening for people with a heavy smoking history remains low. This qualitative interview study of patients and providers from a large ambulatory healthcare system in northern and central California reevaluated perceptions of lung cancer screening with low-dose computed tomography (LCS-LDCT) 5 years after Medicare coverage. We hypothesized that initial attitudes and barriers within the LCS-LDCT discussion and process have likely persisted with little change since Medicare coverage and we sought to understand how these attitudes continue to impact effective implementation and uptake of screening with the goal of identifying opportunities for improvement. Between 2019 and 2020, interviews were conducted with 10 primary care physicians and 30 patients using semi-structured interview guides. Providers and patients expressed that they were both aware and supportive of LCS-LDCT, a change from earlier studies, but continued to report little to no shared decision making nor use of a decision aid despite being Medicare requirements. Creation and incorporation of a single-page, graphic heavy decision aid may help address many of the persistent barriers around implementation for both providers and patients. Given recently expanded guidelines from the U.S. Preventive Services Task Force for LCS-LDCT screening and their coverage by Medicare, it is important for healthcare systems to understand provider and patient perceptions to further improve the implementation of LCS-LDCT to ultimately reduce lung cancer mortality.

**Supplementary Information:**

The online version contains supplementary material available at 10.1186/s12875-022-01925-2.

## Introduction

Lung cancer is the leading cause of cancer-related deaths for both men and women in the United States [1]. Typically, lung cancer is diagnosed late in the disease process when the 5-year survival rate is only 7% [2]. In 2011, results from the National Lung Cancer Screening Trial (NLCST) showed a 20% relative decrease in lung cancer mortality using low-dose computed tomography (LDCT) compared to chest X-rays for those individuals at high-risk who currently or formerly smoked cigarettes [3], and as a result, lung cancer screening (LCS) with LDCT was recommended to people who smoke heavily by the U.S. Preventive Services Task Force (USPSTF) in 2013 [4], covered by Medicare in 2015 [5], and eligibilty criteria expanded in 2021 [6]. As of 2020, LCS-LDCT rates remain low with only 3.9% to 7.3% of all eligible individuals having ever received a scan [6–8]. Furthermore, this range varies significantly by state, with Massachusetts having a rate of 17.8% and California and Wyoming having only 1.0% [6]. Given that the eligible population increases significantly under the newly expanded guideline criteria, these LCS-LDCT uptake rates may now be even lower.

Given the low uptake of LCS-LDCT, significant barriers exist for patients and providers in the integration of LCS-LDCT into clinical practice beyond just insurance coverage. LCS-LDCT is unique from other preventive cancer screenings (e.g. mammography, colonoscopy) in that it is not targeted to all healthy people on the basis of age. As of 2021, to be eligible for LCS-LDCT and covered by Medicare one must: 1. be between 50 and 77 years old, 2. currently smoke or quit within the past 15 years, and 3. have a 20-pack-year smoking history [9]. Furthermore, LCS-LDCT is the only preventive screening test which Medicare requires documentation of tobacco cessation counseling and a shared decision making (SDM) encounter using one or more decision aids for reimbursement [9]. Previous qualitative studies have suggested that the SDM and counseling requirement may be onerous for providers within an already busy clinical visit and may be impacting LCS-LDCT implementation and uptake [10, 11].

In addition, these people who have smoked long-term are an under-served group due to factors including shame over smoking, feelings of victim blaming that they caused their own troubles, fear of diagnosis, and distrust of the healthcare system [12, 13]. For lung cancer screening specifically, patients have cited barriers of inconvenience, distrust, and stigma [13], in addition to lack of awareness, cost, fear of radiation exposure, socioeconomic barriers, and access [14]. Previous interview studies with providers have reported barriers including unfamiliarity with LCS guidelines, challenges with eligibility criteria, and skepticism regarding the evidence [11, 15, 16].

These barriers make utilization of LCS-LDCT in healthcare systems challenging. In order to increase lung cancer screening more widely in the U.S., it is critical to understand perceptions of LCS-LDCT at the patient and provider levels to identify and address modifiable barriers for LCS-LDCT. Most previous work has been conducted in health care settings like Veterans Health Administration [10, 11, 17, 18], community health clinics/federally-qualified health centers/urban safety-net clinics [17, 19, 20], and university health centers [10, 13], but only a few interview studies have explored how a large sample of both providers and patients within the same healthcare system describe LCS-LDCT [11, 17, 21, 22]. We interviewed providers and patients within a large ambulatory healthcare delivery system to understand each groups’ experience and identify similarities and differences that may elucidate recommendations for improvements. We hypothesized that initial attitudes and barriers within the LCS-LDCT discussion and process have likely persisted with little change since Medicare coverage and we sought to understand how these attitudes and barriers continue to impact effective implementation and uptake of screening with the goal of identifying opportunities for improvement.

## Methods

### Setting and recruitment

The study was conducted at Sutter Health, a large, multispecialty healthcare delivery system in northern and central California that serves approximately 3.5 million patients across 22 counties and is representative of the underlying catchment area: 45.6% non-Hispanic white, 15.6% Hispanic, 16.5% non-Hispanic Asian, 4.7% non-Hispanic Black, and 17.4% non-Hispanic other as of 2020 [23]. Sutter Health began offering LCS-LDCT to patients in 2012, and implemented an LCS-LDCT SmartSet (a pre-programed Epic tool to help walk providers through orders and referrals of specific procedures, such as LCS-LDCT) in 2018.

We used semi-structured interviews to qualitatively understand both patient and provider perceptions and/or experience of LCS-LDCT. The interviews were one part of a larger mixed-methods study on the implementation of LCS-LDCT at Sutter Health with results reported elsewhere [8, 24].

Research team members developed the interview guides for both patients and providers based on findings from literature review and the quantitative analyses of the larger study [8, 24]. We found that while LCS-LDCT orders have increased, not all of these orders were appropriate referrals as patients did not meet all the CMS eligibility criteria for LCS. We also noted that very few primary care providers (PCPs) bill for SDM visits or smoking cessation counseling (internal data). Given these findings, we focused our provider interviews on knowledge of LCS-LDCT, attitudes toward screening guidelines, perspectives and experiences using LCS-LDCT as a screening test for lung cancer, tobacco cessation counseling efforts, SDM, and the best ways to help patients make decisions on LCS-LDCT. Patient interviews included questions about awareness of LCS-LDCT, perceptions of pros and cons of LCS-LDCT, experiences with SDM and smoking cessation, and ways to improve physician–patient communication (see Appendix A for interview guides). Questions were finalized after input from our internal Advisory Board (AB) which was comprised of a Sutter Health primary care physician, pulmonologist, and operational leaders. All participants provided written informed consent prior to the start of the interview. All study materials were reviewed and approved by the Sutter Health Institutional Review Board.

For provider interviews, we identified providers with the highest and lowest LCS-LDCT referral/order rates among those who had seen at least 20 LCS-LDCT eligible patients from across the system. To be eligible for an interview, providers had to practice at least 20 hours per week in Sutter Health facilities and have seen at least 20 guideline-eligible patients. We emailed 45 providers initial recruitment information with up to three additional requests. Two researchers (MM and JL) conducted 10, hour-long provider interviews, either in person (6) or over the phone (4) between January and December 2019 and participants received a $150 gift card for their time.

For patient recruitment, potential participants were identified from eligible study providers using electronic health records (EHRs). At the time of the study in 2019 and 2020, the Medicare eligibility guidelines were different in that patients needed to be age 55–80 years old, currently smoke or quit within the past 15 years, and have a 30-pack-year smoking history. It was these earlier guideline criteria that served as our inclusion criteria. We recruited from 3 patient groups: 1. those who received an LCS-LDCT order and completed the screening (Group 1); 2. those who received an LCS-LDCT order but not completed (Group 2); and 3. those who never received an LCS-LDCT order despite evidence of eligibility in the EHR (Group 3). For this analysis, we were focusing on the similarities across all 3 groups rather than on differences that may exist within each group to identify common areas that would allow the creation of interventions to overcome persistent barriers. Additionally, because of interviewer language, patients had to be able to speak and read English to participate in the interview. Patients were sent initial recruitment materials via USPS mail and up to 3 reminder letters. We contacted 282 patients and conducted 30 interviews from those who responded and completed informed consent – 14 in-person and 16 over the phone. Patient interviews were conducted by MM and/or JL between April 2019–December 2020 and lasted 45–60 min. All patients received a $40 gift card for their time. We reached data saturation for categories after 10 provider interviews and 30 patient interviews.

### Analysis

Both patient and provider interviews were audio-recorded and transcribed for accuracy. Qualitative analyses were managed using Dedoose 9.0. We created a codebook using a grounded theory approach which allowed for ideas to emerge from the data. Initial coding reflecting the source data was created and grouped into associated clusters. These clusters and the inter-relationships were summarized into code categories, and the emerging categories, based on the research questions, were determined as a group. The team discussed, addressed, and resolved disagreements for each step of the analysis in a series of meetings. Initial categories were discussed with the wider research team, amended, and renamed until a consensus was reached. Any new data that did not fit into the existing code categories were highlighted and discussed further, with subsequent amendments. Following the compilation and analysis of data, the study team presented the initial results to the AB. Their input and comments were further incorporated to improve the accuracy, completeness, and usefulness of the information in the final analysis. Patient and provider interviews were coded separately, but general categories were aligned to develop a cohesive coding scheme for providers and patients. MM and JL both reviewed and coded text samples to create the initial codebook; MM did all coding; JL and CS reviewed the findings and further validated the coding.

Demographic characteristics for patients and providers were obtained from EHR and administrative data.

## Results

We interviewed 10 providers and 30 patients. Participants’ demographic characteristics are shown in Table 1. The median age of patient participants was 70, and more than half were women, non-Hispanic white, used to smoke, and had Medicare insurance. We interviewed 13 (43%) patients from group 1, 9 (30%) patients from group 2, and 8 (26.7%) patients from group 3. The average age of participating providers was 49.3, and the majority were non-Hispanic white, female, and in Family Medicine.

**Table 1:** Characteristics of patient and provider participants

*Patient Characteristics*	*Number* (*N* = 30)	*Percent*
**AGE**		
Mean age, years	71	N/A
Median age, years	70	N/A
Age range, years	60—87	N/A
**SEX**		
Female	17	56.7%
Male	13	43.3%
**RACE/ETHNICITY**		
African American	1	3.3%
Hispanic/Latino	2	6.7%
White	20	66.7%
Other	2	6.7%
Unknown	5	16.7%
**SMOKING STATUS**		
Currently smokes	4	13.3%
Formerly smoked	26	86.7%
**PRIMARY INSURANCE STATUS**		
Medicare FFS	15	50%
Medicare HMO	7	23.3%
Medicaid/Medi-Cal	2	6.7%
Private	5	16.7%
Unknown	1	3.3%
**PATIENT GROUP**		
Group 1 (received LCS-LDCT order and completed screening)	13	43.3%
Group 2 (received order, but have not completed screening)	9	30%
Group 3 (no order)	8	26.7%
*Provider Characteristics*	*Number* (*N* = 10)	*Percent*
**AGE**		
Mean age, years	49.3	N/A
Median age, years	51	N/A
Age range, years	34 – 62	N/A
**SEX**		
Female	9	90%
Male	1	10%
**RACE/ETHNICITY**		
Non-Hispanic Asian	4	40%
Non-Hispanic white	6	60%
**SPECIALTY**		
Family Medicine	8	80%
Internal Medicine	2	20%

Based on our analyses, we identified 2 main categories of responses – screening awareness and identification of eligible individuals, and patient-provider communication about LCS-LDCT (Table 2)–described in detail below.

**Table 2:** Categories, subcategories, and associated findings

CATEGORY	FINDINGS
**Screening awareness and identification of eligible individuals: difficulties determining eligibility**	
*Provider*	All knew about LCS-LDCT as an option and reported ordering it for eligible patients. Many providers felt that the annual physical was the most appropriate time to offer screening. Providers thought those who currently smoke may show more interest in LCS-LDCT
*Patient*	Most were aware of LCS-LDCT. Many patients felt providers should offer this once a year only at an annual physical or visit. In contrast to provider respondents, patients felt those who formerly smoked would be most receptive to LCS-LDCT
*Provider difficulties with eligibility criteria even after creation of a SmartSet*	Providers find calculating pack-years to be the most difficult element of determining eligibility. Very few providers knew of or used the SmartSet as a tool to aid in LCS-LDCT
**Patient-provider communication about LCS-LDCT**	
*Similar perceived benefits of LCS-LDCT*	Most providers cited the main benefit of LCS-LDCT as early detection, while patients most frequently mentioned the benefits of LCS-LDCT as early detection of lung cancer, that it can support smoking cessation, and impact on patient peace of mind
*Pros outweigh cons for LCS-LDCT*	Perceived risks commonly noted by both providers and patient for LCS-LDCT were radiation and patient psychological distress. And yet, patients and providers both overwhelming felt that pros outweighed all cons of screening
*Shared Decision Making (SDM) limited to brief conversations about risks and benefits*	Providers acknowledged little to no SDM discussion, while no patient could recall one at all, claiming that there was only a little conversation with the provider about risks and benefits
*Providers most influential*	Half of patient respondents felt strongly that the most important factor influencing their decision to do the screening was a physician recommendation
*Use of a decision aid handout would benefit providers and patients*	Providers and patients were supportive of incorporation of a decision aid. Patients liked a handout that could be delivered ahead of an upcoming visit. Providers were warier of this timing as they worried about it creating additional anxiety and instead wanted information to give in an after-visit summary

### Screening awareness and identification of eligible individuals: difficulties determining eligibility

#### Provider

Every provider interviewed said they were aware of LDCT as a screening option for lung cancer and ordered it for eligible patients; however, two did admit to a lack of consistency in their ordering. Providers primarily reported offering screening during annual physicals, wellness visits, and new patient visits, with fewer thinking to suggest it during “appropriate” acute care visits (e.g., for sinus infections or colds) (60% vs. 40%, respectively). Providers were divided on whether those who currently smoke or formerly smoked would be more receptive to screening with 4 believing those who currently smoke show greater interest, 4 feeling there would be no difference, and only 2 believing that those who recently quit smoking would be the most receptive. One provider noted that they focus more on LCS-LDCT for those who currently smoke because it is harder for them to remember to talk about screening with those who formerly smoked.

#### Patient

Seventy three percent of patient participants (22 out of 30) said they were aware of LCS-LDCT. Of those who offered an opinion on how frequently LCS-LDCT should be brought up and discussed by a provider, 15 felt once a year at the physical was appropriate, while 9 though it should be mentioned at any visit. One patient strongly opposed having it brought up at every visit saying: “I think the doctor’s primary objective is to present at annual physical…but to do it at every visit, because I’ve got to come in and see her for my diabetes…I’ll find another doctor…some people like to get beat over the head with a baseball bat. Not me” (Patient 1). Opposite to physician respondents, of those who answered this question, 14 patients felt those who formerly smoked would be most receptive to LCS-LDCT, while 12 thought there would be no difference between those who currently smoke or formerly smoked, and only 2 thought those who currently smoke would be most receptive.

#### Provider difficulties with eligibility criteria even after creation of a SmartSet

Because of the multiple criteria for LCS-LDCT, providers noted that they frequently had to take the time to look up eligibility criteria before recommending LCS-LDCT to patients. A commonly cited difficulty was the discrepancy between the upper age limit allowed by the USPSTF and Medicare – 80 vs. 77. Physicians noted the difficulty in calculating pack years and how this complicated the screening process: “I think the part where this becomes a little subjective is the way you decide to document how many pack years they have. And I think that’s rarely accurate, and since it matters a lot for this, that’s one of the things that gives me a little bit of pause” (Provider 1). Respondents cited difficulties with the integration of LCS-LDCT ordering into the current EHR system noting that key variables, e.g., smoking history, are often missing or inaccurate. Furthermore, despite creation of a “SmartSet” in April 2018 within the EHR to help walk providers through the process of an LCS-LDCT order, only 4 provider participants were aware of and used the SmartSet option at the time of the interview in 2019.

### Patient-provider communication about LCS-LDCT

#### Similar perceived benefits of LCS-LDCT

Most providers cited the main benefit of LCS-LDCT as early detection, while patients most frequently mentioned the benefits of LCS-LDCT as early detection of lung cancer, that it can support smoking cessation, and impact on patient peace of mind. One patient noted how LCS-LDCT helped with their cessation, saying “this is a good boot in the butt, telling me I’ve only stop smoking 20-some days, but [I’m] not going to pick up another cigarette” (Patient 8). Additionally, a provider noted that in his experience among patients who had undergone LCS-LDCT there was a higher rate of smoking cessation, regardless of results. Regarding peace of mind, six patients expressed that they “wanted to know” the scan findings so they could either “move on with my life or…put a stop to [worrying about cancer]” (Patients 2 and 3, respectively).

#### Pros outweigh cons for LCS-LDCT

Perceived risks commonly noted by both providers and patient for LCS-LDCT were radiation and patient psychological distress. Providers and patients frequently expressed concern with radiation from CT scans being “a significant exposure to risk for cancer,” (Provider 7) especially annually as recommended for LCS. However, some providers did often temper their concerns about radiation by noting how low the exposure was, especially relative to other CT scans and the poor quality of chest x-rays: “I think the benefit of the low dose CT is much better than the prior screening with chest x-rays, and I think the emphasis is on the significantly lower radiation compared to a routine CT. I think the benefit of the CT and maybe slightly increased risk of radiation does outweigh the risk of the poor quality of the chest x-ray.” (Provider 6). One provider also noted that the harms of smoking likely outweigh any radiation concerns – “I think the radiation consideration, they probably think about it, but honestly, if they’re already smoking, I don’t think they worry about the little bit of radiation that they would get” (Provider 4).

Six of the 10 providers also commented about the scan causing additional psychological distress for patients: “It’s not easy to counsel people on whether or not to have something worked up once you find something small. They always get anxious and you try to make your best guess” (Provider 2). One provider likened LCS-LDCT to other preventive cancer screenings saying, “I think that some of the challenges is that, for really any health maintenance screening, is they’re worried about what it’s going to find. So, I think the fear is there. That’s why people don’t get mammograms done or colonoscopies, sometimes” (Provider 4). Almost half of patients also felt the psychological distress related to the screening, particularly the high rates of “false alarms,” would be difficult and may instead cause some people to forego the scan and “put [their] head in the sand” (Patient 4). A few providers expressed some frustration at the number of false positives vs. true positives with this screening – “I’ve probably been doing [screenings] for the last two years or so. I think there’s coming up to be more cons or more risks with the…false positives than the true positives in patients because the ones with lung cancer, which we found ourselves, are people who are symptomatic…from the lung cancer screening I think there’s more…false positives” (Provider 2). And yet, despite these perceived risks, 4 providers and 26 patients specifically mentioned that they felt the pros of screening outweighed any cons.

#### Shared Decision Making (SDM) limited to brief conversations about risks and benefits

SDM is a Medicare requirement for coverage. All 10 physicians said they do SDM and LCS counseling as part of the regular patient visit and that it was typically a fairly short discussion: “it doesn’t take a long time” and “around, you know, five minutes or something like that” (Provider 3). Given these very brief discussions, a Family Medicine provider admitted that patients “probably [did] not” understand all the pros and cons of screening” (Provider 4).

Interestingly, when asked explicitly during the interview, no patient said they had a SDM discussion with a provider, and most patients said there was only a little conversation with the provider about risks and benefits. In fact, a few patients were not being aware of the benefits of LCS-LDCT screening when asymptomatic:

*For people that don’t have any symptoms and they smoke?...Hmm, no, I don’t think so. Nuh-uh…Because they say it takes a year to have your lungs go back to normal after smoking. And if they’re—you know, not coughing, nothing like that? No. I wouldn’t. Uh-uh. (Patient 5)*

#### Providers most influential

Half of patient respondents felt strongly that the most important factor influencing their decision to do the screening was a physician recommendation. One patient expressed – “Who more are you going to have confidence in if it’s not your doctor…just the authority that she says, ‘You’re eligible for this, you should have it done’” (Patient 3). This trust in physicians was also mentioned by another patient: “Well, definitely having it suggested by your doctor. Because the doctors generally know what’s the right thing to do” (Patient 6). A third patient suggested that this type of screening is only likely to happen if the physician mentions it: “…the doctor talking to them…because that’s usually what causes people to have tests and that sort of thing…because an ordinary person just doesn’t on their own say, ‘Oh, gee, I think I should go get my lungs scanned’” (Patient 7).

#### Use of a decision aid handout would benefit providers and patients

Providers and patients all felt regular use of a decision aid in LCS-LDCT would be beneficial for both parties. We asked specifically about the distribution and design of a decision aid to provide to patients. Participants overwhelmingly supported presenting a decision aid as a handout (as opposed to electronically). When asked about sending decision aid materials in advance of an upcoming visit providers noted two specific concerns: 1. accurately identifying patients eligible for LCS-LDCT using EHR data is extremely difficult, and 2. providing information on LCS-LDCT can potentially be interpreted by patients as saying “you might have cancer, you might die” and is “anxiety-provoking stuff,” (Provider 5) so there would need to be somebody immediately available to talk to. Given these concerns, providers overall were very interested in having information on LCS-LDCT included as part of a patient’s After Visit Summary (AVS). In contrast, half of patients thought receiving materials ahead of an upcoming visit may provide more time to review and reflect before talking with their doctor.

Patients recommended that the decision aid could explain how the CT scan differs from an MRI, helping those patients who are concerned with being “stuck in a little tube,” (Patient 7) a refrain mentioned among our respondents. A few patients also felt including information on the signs and symptoms of lung cancer was important, as many admitted to not knowing what these were. Other suggestions from patient respondents to be included in decision aids were additional information on risks and benefits, expectations (that it is a yearly screening), that it is covered by Medicare, eligibility criteria, and effective smoking cessation resources. As one patient mentioned when commenting on the benefit of a decision aid: “…there are benefits…and the risks are less than…you might think…once they start considering it, it just makes a lot of sense. And it’s a rational choice, but so when you realize that it is a rational choice, you realize that not doing it is more irrational” (Patient 9). Graphics could help greater understanding, particularly for those with lower education, and font-size variation and color would draw the eye and help with interest in the materials. Importantly, patients felt that the length would need to be short, many suggesting that one page would be ideal. Patients also suggested a website with more information would be a way to include more details for those interested without adding length to the handout.

## Discussion

In this interview study of patients and providers from a large healthcare system in northern and central California, we found that both groups overwhelmingly reported awareness of and support for LCS-LDCT. This is different from lower rates of previous studies that found only 25% to 40% of patients may have knowledge or awareness of LCS-LDCT [25, 26]. And yet despite the reported high awareness from our patient and provider participants, overall screening rates across Sutter Health continue to hover at 7% [8], similar to rates across the U.S. This presents added complexity as others have pointed to knowledge and awareness as a barrier to LCS-LDCT uptake [15, 26], yet our findings suggest that knowledge and awareness of LCS-LDCT alone are not sufficient to increase screening rates.

Similar to previous studies [27, 28], our provider respondents continued to report difficulty in accurately identifying eligible patients despite an existing SmartSet within the organization’s EHR. Given the limited reported uptake with the SmartSet, an alternative could be incorporating automatic annual health maintenance reminders for LCS-LDCT eligible patients into the EHR like other preventive screening tests such as colonoscopy or mammogram [29–32]. Half of patients and 60% of providers agreed that the annual physical was the appropriate place to discuss lung cancer screening. Thus, these annual EHR alerts could reduce some of the mental burden required of physicians by indicating a patient may be eligible for screening [33], particularly for those who formerly smoked. Creating alerts and reminders within the EHR for physicians to regularly discuss LCS-LDCT may alone significantly increase LCS-LDCT uptake, especially given that provider recommendation and patient-provider communication have been shown to be strongly influential in screening uptake for other types of preventive cancer screenings [34–38], and was noted to be the most significant driver to LCS-LDCT among our patient participants.

Despite being a requirement for Medicare coverage, providers and patients reported that SDM was brief and mainly limited to risks and benefits during the encounter, suggesting that SDM has still not been well-integrated into the LCS process [21, 39]. These limited discussions may leave patients with unanswered questions and may in part be why there are still low rates of LCS-LDCT screening as they might not understand enough about the pros and cons of screening to be able to make a “preference-sensitive” decision.

Patients and providers in our study continue to report the same benefits and concerns of LCS-LDCT as have been reported previously [15, 19, 40], which was not entirely surprising given that it can take up to 17 years to translate clinical evidence into practice [41]. It is interesting to note that knowing the findings from test results was noted by patients as both a benefit providing “peace of mind” and also a risk of causing undue psychological distress, highlighting the double-edged nature of LCS which may impact screening rates. While it is understandable that patients would want to know their cancer status, LCS-LDCT is based on a high-risk behavior and may be adversely affected by the stigma attached to those who smoke. So, unlike other preventive cancer screening tests where patients are mainly screened based on age, LCS-LDCT may bring feelings like guilt and regret of possibly causing the cancer, thus creating additional psychological distress [12, 13]. That these remain unchanged over time indicates that providers may want to use the SDM discussions to focus specifically on the benefits of early detection, regardless of smoking status, noting that it is always better to find and treat cancer early even for those who currently smoke.

We found that both patients and providers were overwhelmingly supportive of a decision aid which suggests that incorporating one could potentially improve screening rates. Patients expressed that the decision aid should be thoughtfully designed, as a single page handout with color and graphics conveying risks and benefits. However, more research is necessary to determine the timing of when to distribute the decision aid as we found differences between patients and providers. Previous research has shown that providers worry about patients engaging in endless internet searching or coming to hold false beliefs about LCS-LDCT before the clinical visit [42] which could explain why our provider participants would rather have the decision aid included in the AVS. Whereas our patient respondents’ recommendation of providing a decision aid ahead of time may ease the burden on providers to remember to recommend the screening test and may put the onus more on patients to bring it up if interested. Regardless of the timing of decision aid distribution, it is unclear what the actual impact of a decision aid might be given that our patient participants expressed that the most influential factor in their decision was a provider recommendation. Thus, further research is necessary to ultimately see what has greatest impact–using a decision aid or an explicit provider recommendation, or a combination of both.

We recognize several limitations with the current study. First, all participants came from one healthcare system in northern and central California, were primarily non-Hispanic white, and patients were well-insured, which may not be representative of providers and patients in other areas of the U.S. Second, all interviews were conducted in English; it may be that non-English speakers have different perceptions and barriers from those presented in this interview study. Third, most patients interviewed were those who formerly smoked who had completed at least one scan. Individuals who currently smoke and patients who opt out after a referral from a provider may have differing perspectives on the value or importance of LCS-LDCT. Additionally, most providers interviewed identified as female; it is possible that male providers may have different experiences and perceptions. Finally, we did not have any patients on our advisory board, and their contributions could have differently shaped the study design, methods, interpretation of results, and dissemination of findings. Future studies should include patients on the advisory board to help reach a more diverse population of respondents to confirm that our results are consistent across provider and patient groups.

## Conclusion

Given that uptake of LCS-LDCT continues to remain low, it is important to understand both provider and patient perceptions in real-world implementation of LCS to identify modifiable components and create more targeted interventions to increase utilization rates to detect lung cancer early in the disease course. We found that while awareness of LCS-LDCT has increased, little has changed in the years since initial Medicare coverage of LCS-LDCT, which suggests that other interventions for both patients and providers are necessary to increase screening rates such as a single-page, graphic heavy decision aid or scheduling automatic annual screening alerts in the EHR.

## Supplementary Information

**Additional file1:** **Appendix A.** Provider and patient interview guides.

## Data Availability

The datasets analyzed during the current study may be available from the corresponding author on reasonable request.

## References

[1] Centers for Disease Control and Prevention. An Update on Cancer Deaths in the United States. US Department of Health and Human Services, Centers for Disease Control and Prevention, Division of Cancer Prevention and Control. https://www.cdc.gov/cancer/dcpc/research/update-on-cancer-deaths/index.htm. Published 2021. Accessed 28 July 2021.

[2] National Cancer Institute. Cancer Stat Facts: Lung and Bronchus Cancer. https://seer.cancer.gov/statfacts/html/lungb.html. Published 2000. Accessed 7 Feb 2022.

[3] Kramer BS, Berg CD, Aberle DR, Prorok PC (2011). Lung cancer screening with low-dose helical CT: results from the National Lung Screening Trial (NLST). J Med Screen.

[4] Moyer VA (2014). U.S. Preventive Services Task Force. Screening for Lung Cancer: U.S. Preventive Services Task Force Recommendation Statement. Ann Intern Med.

[5] Centers for Medicare & Medicaid Services. Screening for lung cancer with Low Dose Computed Tomography (LDCT). https://www.cms.gov/medicare-coverage-database/view/ncacal-decision-memo.aspx?proposed=N&NCAId=274. Accessed 7 Feb 2022.

[6] American Lung Association State of Lung Cancer. Lung cancer key findings. https://www.lung.org/research/state-of-lung-cancer/key-findings#:~:text=receive%20no%20treatment.-,Nationally%2C%20only%2024%25%20of%20cases%20are%20diagnosed%20at%20an%20early,eligible%20were%20screened%20in%202020. Accessed 2 Nov 2022.

[7] Jemal A, Fedewa SA (2017). Lung Cancer Screening With Low-Dose Computed Tomography in the United States—2010 to 2015. JAMA Oncol.

[8] Li J, Chung S, Wei EK, Luft HS (2018). New recommendation and coverage of low-dose computed tomography for lung cancer screening: uptake has increased but is still low. BMC Health Serv Res.

[9] Medicare.gov. Lung cancer screenings. https://www.medicare.gov/coverage/lung-cancer-screenings. Accessed 12 June 2022.

[10] Melzer AC, Golden SE, Ono SS, Datta S, Crothers K, Slatore CG (2020). What Exactly Is Shared Decision-Making? A Qualitative Study of Shared Decision-Making in Lung Cancer Screening. J Gen Intern Med.

[11] Kanodra NM, Pope C, Halbert CH, Silvestri GA, Rice LJ, Tanner NT (2016). Primary Care Provider and Patient Perspectives on Lung Cancer Screening. A Qualitative Study. Ann Am Thorac Soc.

[12] Chapple A, Ziebland S, McPherson A (2004). Stigma, shame, and blame experienced by patients with lung cancer: qualitative study. BMJ.

[13] Carter-Harris L, Ceppa DP, Hanna N, Rawl SM (2017). Lung cancer screening: what do long-term smokers know and believe?. Health Expect.

[14] Jonnalagadda S, Bergamo C, Lin JJ (2012). Beliefs and attitudes about lung cancer screening among smokers. Lung Cancer.

[15] Wang GX, Baggett TP, Pandharipande PV (2019). Barriers to Lung Cancer Screening Engagement from the Patient and Provider Perspective. Radiol.

[16] Ahsan A, Zimmerman E, Rodriguez EM (2019). Examining Lung Cancer Screening Behaviors in the Primary Care Setting: A Mixed Methods Approach. J Cancer Treat Res.

[17] Wiener RS, Koppelman E, Bolton R (2018). Patient and Clinician Perspectives on Shared Decision-making in Early Adopting Lung Cancer Screening Programs: a Qualitative Study. J Gen Intern Med.

[18] Gesthalter YB, Koppelman E, Bolton R (2017). Evaluations of Implementation at Early-Adopting Lung Cancer Screening Programs: Lessons Learned. Chest.

[19] Hoffman RM, Sussman AL, Getrich CM (2015). Attitudes and Beliefs of Primary Care Providers in New Mexico About Lung Cancer Screening Using Low-Dose Computed Tomography. Prev Chronic Dis.

[20] Mishra SI, Sussman AL, Murrietta AM (2016). Patient Perspectives on Low-Dose Computed Tomography for Lung Cancer Screening, New Mexico, 2014. Prev Chronic Dis.

[21] Brenner AT, Malo TL, Margolis M (2018). Evaluating Shared Decision Making for Lung Cancer Screening. JAMA Intern Med.

[22] Simmons VN, Gray JE, Schabath MB, Wilson LE, Quinn GP (2017). High-risk community and primary care providers knowledge about and barriers to low-dose computed topography lung cancer screening. Lung Cancer.

[23] Azar KMJ, Lockhart SH, Shen Z (2021). Persistence of Disparities Among Racially/Ethnically Marginalized Groups in the Coronavirus Disease 2019 Pandemic Regardless of Statewide Shelter-in-Place Policies: An Analysis From Northern California. Am J Epidemiol.

[24] Li J, Chung S, Martinez MC, Luft HS (2020). Smoking-Cessation Interventions After Lung Cancer Screening Guideline Change. Am J Prev Med.

[25] Williams LB, Looney SW, Joshua T, McCall A, Tingen MS (2021). Promoting Community Awareness of Lung Cancer Screening Among Disparate Populations: Results of the cancer-Community Awareness Access Research and Education Project. Cancer Nurs.

[26] Percac-Lima S, Ashburner JM, Atlas SJ, Rigotti N, Poles E, Park ER (2016). Beliefs about lung cancer, knowledge, and interest in lung screening among community health center patients. J Clin Oncol.

[27] Kinsinger LS, Anderson C, Kim J (2017). Implementation of Lung Cancer Screening in the Veterans Health Administration. JAMA Intern Med.

[28] Zeliadt SB, Hoffman RM, Birkby G (2018). Challenges Implementing Lung Cancer Screening in Federally Qualified Health Centers. Am J Prev Med.

[29] Yabroff KR, Zapka J, Klabunde CN (2011). Systems Strategies to Support Cancer Screening in U.S. Primary Care Practice. Cancer Epidemiol Biomarkers Prev.

[30] Gandhi TK, Sequist TD, Poon EG (2003). Primary care clinician attitudes towards electronic clinical reminders and clinical practice guidelines. AMIA Annu Symp Proc.

[31] Kern LM, Barrón Y, Dhopeshwarkar RV, Edwards A, Kaushal R, with the HI (2013). Electronic Health Records and Ambulatory Quality of Care. J Gen Intern Med.

[32] Shea S, DuMouchel W, Bahamonde L (1996). A Meta-analysis of 16 Randomized Controlled Trials to Evaluate Computer-Based Clinical Reminder Systems for Preventive Care in the Ambulatory Setting. J Am Med Inf Assoc.

[33] Peterson E, Harris K, Farjah F, Akinsoto N, Marcotte LM (2021). Improving smoking history documentation in the electronic health record for lung cancer risk assessment and screening in primary care: A case study. Healthcare.

[34] Dominick KL, Skinner CS, Bastian LA, Bosworth HB, Strigo TS, Rimer BK (2003). Provider Characteristics and Mammography Recommendation among Women in Their 40s and 50s. J Women's Health.

[35] Ye J, Xu Z, Aladesanmi O (2009). Provider recommendation for colorectal cancer screening: Examining the role of patients’ socioeconomic status and health insurance. Cancer Epidemiol.

[36] Ramdass P, Petraro P, Via C, Shahrokni A, Nawaz H (2014). Providers Role in Colonoscopy Screening for Colorectal Cancer. Am J Health Behav.

[37] Peterson EB, Ostroff JS, DuHamel KN (2016). Impact of provider-patient communication on cancer screening adherence: A systematic review. Prev Med.

[38] National Institutes of Health State-of-the-Science Conference Statement (2010). Enhancing Use and Quality of Colorectal Cancer Screening. Ann Intern Med.

[39] Carter-Harris L, Tan ASL, Salloum RG, Young-Wolff KC (2016). Patient-provider discussions about lung cancer screening pre- and post-guidelines: Health Information National Trends Survey (HINTS). Patient Educ Couns.

[40] Ersek JL, Eberth JM, McDonnell KK (2016). Knowledge of, attitudes toward, and use of low-dose computed tomography for lung cancer screening among family physicians. Cancer.

[41] Morris ZS, Wooding S, Grant J (2011). The answer is 17 years, what is the question: understanding time lags in translational research. J R Soc Med.

[42] O'Mathuna DP (2018). How Should Clinicians Engage With Online Health Information?. AMA J Ethics.

