# Supplementary material for: Provider and patient perspectives to improve lung cancer screening with low-dose computed tomography 5 years after Medicare coverage: a qualitative study
Source: BMC Prim Care. 2022 Dec 20;23:332. doi: 10.1186/s12875-022-01925-2 (PMC9768892; doi:10.1186/s12875-022-01925-2)
Supplement: Supplementary file 1 — Additional file1: Appendix A. Provider and patient interview guides. [file 12875_2022_1925_MOESM1_ESM.docx]

**APPENDIX A**

***Provider Interview Guide***

**Opening**

- Introduction and purpose of the interview. (*Study to understand challenges of patients and providers in implementing lung cancer screening and smoking cessation counseling).*

- Explain what will happen to the information received. (*Confidential; we will not share our notes with anyone; they will become integrated into reports without personal attribution.).*

- Discuss audio-recording and obtain permission. (*Audio-recording is to remember accurately what was conveyed, without adding our own interpretation to your statements*).

- Sign informed consent.

**I. Lung Cancer Screening**

1. I’d like to start by first hearing about your thoughts on lung cancer screening (LCS) with low dose CT (LDCT). What have you heard about these recent developments? Do you have any thoughts or opinions about the pros/cons of LDCT screening with your patients?
   1. Do you currently screen heavy smokers for lung cancer? Do you know who pays for that?
   2. Is there a formal counseling visit before referring your patients to LCS? How do you communicate with your patients in order to make a shared decision? (e.g., talk about risks and benefits of LCS? provide your patients choices and options in regard to their concurrent conditions? convey confidence in patients’ ability to make a right decision? listen to patients, understand how they would like to do things before making a referral to LCS, and encourage them to ask questions?)

***[Interviewer to review FAQ sheets--NLST Summary and Guideline Fact Sheets (ACS, ALA, USPSTF)*** I have some information about some of the research that has led to current screening recommendations as well as initial guidelines that I would like to briefly review with you.**]**

1. What are your thoughts about this information and screening recommendations?

Prompts: I would like to focus on a few specific areas with you

- - 1. What are your views about the relative risks and benefits to LDCT screening for your patients?

*(Possible risks: fear, radiation exposure, false positives/incidental findings, over diagnosis, complications from diagnostic follow up)*

*(Possible benefits: enhance tobacco cessation efforts, reduce lung cancer deaths, peace of mind)*

- - 1. Thinking from the perspective of providers like you, how receptive would they be to implementing LCS? How feasible is this for you? What are challenges to refer patients to LCS? What would help encourage providers like you to refer patients to LCS?
    2. Private insurance plans cover lung cancer screening for eligible patients age 55 through 80, with no out-of-pocket costs. Medicare pays for lung cancer screening with no out-of-pocket costs for eligible patients age 55 up to 77. There may be additional costs for follow-up tests and/or treatments after the initial screening exam. The patient will have to contact his/her insurance company to find out. Do you have any concerns about the financial burden for patients?

**II. Tobacco Cessation Counseling Efforts**

1. We’re going to talk some more about how best to offer LDCT screening to your patients but before we do that, I’d like to take a step back and hear about your current tobacco cessation counseling efforts. Can you tell me what leads you to spend time with a patient to counsel them to stop smoking?
   - 1. Are you aware of the Surgeon General guidelines and are you familiar with them?
     2. What strategy(ies) have you found to be most effective? (*probe whether medications have been effective)*
     3. What tools, resources, information do you have available to support smoking cessation efforts?
     4. Do you involve other providers/staff in these efforts? Referral to community based programs?
2. In your experience, what leads smokers to stop smoking? *(Possible influences: family/friend; proximity to someone close diagnosed with lung disease/cancer; individual symptoms, cost, etc…)*
   - 1. What or who has the most influence on motivating patients to quit?
     2. Are there any cultural beliefs related to reasons why they might smoke or their efforts to quit smoking?

**III. Putting it all together: LDCT Screening and Tobacco Cessation Shared Decision Making**

1. Thinking about your current tobacco cessation counseling efforts, I would like to hear about what influence you think LCS might have on eligible patients (55-80; 30 pack years, etc…)?
   - 1. How would you integrate LCS into your efforts to help patients to stop smoking?
     2. Would you anticipate different levels of receptivity of LCS with LDCT from either current smokers or those that quit but are still eligible for LCS with LDCT?
     3. Should the provider be the one discussing LCS with LDCT with patients? What about other staff? When should it be brought up (e.g., any visit or only the preventive visits)? How receptive are you to a coordinator taking care of this?

1. An important goal of our project is to help both patients and providers by developing aids to assist with decision making for LCS with LDCT. As we’ve discussed, LCS with LDCT is recommended for patients who meet certain criteria but ultimately it’s up to them. Some people refer to these as “preference sensitive decisions.” What is the best way to help patients make decisions in these situations?
2. Thinking more about decision aids, what information or resources would be most helpful to you and your patients (probe whether length, contents including eligibility criteria, overview of LCS, benefits, risks, smoking cessation information, value clarification, list of questions to talk about at the visit with the health care professional, list of approved LCS centers, and information on insurance coverage would be most important)?
   1. In what format—written brochures/pamphlets, CD, video, web-based, other?
   2. When should these decision aids be distributed and discussed with patients (*probe* whether providing eligible patient a decision aid prior to a visit is a good idea)?

**Closing the interview**

- Thank you for your time and participation!

***Patient Interview Guide***

**Opening**

- Introduction and purpose of the interview. (*Study to understand challenges of patients and providers in implementing lung cancer screening and smoking cessation counseling).*

- Explain what will happen to the information received. (*Confidential; we will not share our notes with anyone; they will become integrated into reports without personal attribution.).*

- Discuss audio-recording and obtain permission. (*Audio-recording is to remember accurately what was conveyed, without adding our own interpretation to your statements*).

- Sign informed consent.

**I. Lung Cancer Screening**

1. I’d like to start by first hearing about your thoughts on lung cancer screening (LCS) with low dose CT (LDCT). What have you heard about it? Do you have any thoughts or opinions about the pros and cons of lung cancer CT screening?
   1. Have you heard about, talked with your health care providers, or received LCS with LDCT?
   2. During your visit, do you feel that your health care providers have talked about risks and benefits of LCS? Did s/he provide you choices and options in regard to your overall health? Did s/he convey confidence in your ability to make a right decision? Did s/he listen to you, understand how you would like to do things before making a referral to LCS, and encourage you to ask questions?

***[Interviewer to review patient information handouts*** I have some information about current screening recommendations that I would like to briefly review with you.**]**

1. What are your thoughts about this information and screening recommendations?

Prompts: I would like to focus on a few specific areas with you

- - 1. How do you feel about the risks and benefits of LCS for yourself? Do you think other people would feel the same about the risks and benefits? Why or why not?

*(Possible risks: fear, radiation exposure, false positives/incidental findings, over diagnosis, complications from diagnostic follow up)*

*(Possible benefits: enhance tobacco cessation efforts, reduce lung cancer deaths, peace of mind)*

- - 1. Do you think other patients similar to you would be willing to consider LCS? Why or why not? How feasible is to complete LCS for you? Did a doctor send you to get LCS? If so, have you done it? What are challenges to getting screened? What would help encourage patients like you to get screened?
    2. Do you have any concerns about the screening procedure or understanding of the results?
    3. Private insurance plans cover lung cancer screening for eligible patients age 55 through 80, with no out-of-pocket costs. Medicare pays for lung cancer screening with no out-of-pocket costs for eligible patients age 55 up to age 77. There may be additional costs for follow-up tests and/or treatments after the initial screening exam. The patient will have to contact his/her insurance company to find out. Do you have any concerns about the financial burden?

**II. Tobacco Cessation Efforts**

1. We’re going to talk some more about how best to offer LCS to patients but before we do that, I’d like to take a step back and hear about your current experiences on quitting smoking. Can you tell me what made you stop smoking (or what would help you to quit? if the interviewee did not quit yet)?
   - 1. What strategy(ies) have you found to be most effective? (*probe whether medications have been effective)*
     2. Are you aware of any tools, resources, information to support smoking cessation?
     3. Have your health care providers provided Tobacco Cessation Counseling to you or referral to community based programs? If yes, what are these programs?
     4. What else can we do to help you quitting smoking?
2. In your experience, what leads patients to stop smoking? *(Possible influences: family/friend; proximity to someone close diagnosed with lung disease/cancer; individual symptoms, cost, etc…)*
   - 1. What or who has the most influence on motivating patients to quit?
     2. Are there any cultural beliefs related to reasons why they might smoke or their efforts to quit smoking?

**III. Putting it all together: LCS and Tobacco Cessation Shared Decision Making**

1. Thinking about your current tobacco cessation efforts, I would like to hear about what influence you think LCS might have on eligible patients (55-80; 30 pack years, etc…)?
   - 1. Are there ways you can think of to make LCS a teachable moment to help patients stop smoking?
     2. Would you anticipate different levels of receptivity of LCS with LDCT from either current smokers or those that quit but are still eligible for LCS with LDCT?
     3. Do you think your physician should be the one discussing LCS with you? What about other staff (like a nurse or medical assistant)? When should it be brought up (e.g., any visit or only the preventive visits)? Sutter is considering using someone to guide patients through the screening process. This person would call to schedule appointments, ensure that you’ve completed all required items before your screening, call about follow-up, etc. Would you be open to having someone like this help you with LCS?)?

1. An important goal of our project is to help both patients and providers by developing aids to assist with decision making for LCS with LDCT. As we’ve discussed, LCS with LDCT is recommended for patients who meet certain criteria but ultimately it’s up to them. Some people refer to these as “preference sensitive decisions.” What is the best way to help patients make decisions in these situations?
2. Thinking more about decision aids, what information or resources would be most helpful to you (probe whether length, contents including eligibility criteria, overview of LCS, benefits, risks, smoking cessation information, value clarification, list of questions to talk about at your visit with your health care professional, list of approved LCS centers, and information on insurance coverage would be most important)?
   1. In what format—written brochures/pamphlets, CD, video, web-based, other?
   2. When do you think this information should be provided to patients (*probe* whether providing eligible patient a decision aid prior to a visit is a good idea)?

**Closing the interview**

- Thank you for your time and participation!
